# Should we routinely perform sperm DNA fragmentation testing in fertility clinics?

**DOI:** 10.1093/hropen/hoag047

**Published:** 2026-06-04

**Authors:** Srisailesh Vitthala, Tarek El-Toukhy, Abha Maheshwari

**Affiliations:** MMC IVF Dubai, Dubai Healthcare City, Dubai, United Arab Emirates; Aberdeen Centre for Reproductive Medicine (ACRM), University of Aberdeen, Aberdeen, UK; Assisted Conception Unit, Guy’s and St Thomas’ Hospital NHS Trust, London, UK; Aberdeen Fertility Centre, NHS Grampian, Foresterhill, Aberdeen, UK

**Keywords:** sperm DNA fragmentation, male infertility, semen analysis, diagnostic accuracy, prognostic testing, screening tests, reproductive outcomes

## Abstract

Routine semen analysis provides limited diagnostic and prognostic insight into male reproductive potential, contributing to the increasing use of sperm DNA fragmentation (SDF) testing in fertility evaluation. However, the clinical application of SDF testing appears to outpace the strength and consistency of the supporting evidence. This opinion paper critically appraises SDF testing through established screening, diagnostic, and prognostic test-performance frameworks, drawing on published observational studies, meta-analyses, and international guideline statements across natural conception and ART. The available evidence indicates that SDF testing does not meet the criteria for population-level screening or for routine diagnostic or prognostic use in unselected infertile populations and is frequently applied beyond its validated scope. Interpretation is further limited by assay heterogeneity, lack of standardization, variable thresholds, reliance on surrogate outcomes, and the limited effectiveness of available interventions. Routine implementation therefore risks overdiagnosis, unnecessary interventions, and premature escalation to assisted reproduction without proven benefit. When applied selectively within an aetiology-driven framework, particularly in defined subgroups, SDF testing may provide adjunctive risk stratification rather than deterministic clinical guidance. All stakeholders must be made aware of these limitations before the test is offered, interpreted, or used to modify treatment.

## Introduction

Male infertility accounts for nearly half of all infertility cases; however, its assessment continues to depend primarily on standard semen analysis as outlined by the WHO laboratory manual (World Health Organization, 2021). Although semen analysis remains the cornerstone of male fertility assessment, its clinical value is fundamentally constrained by its inability to assess sperm functional competence, namely, the capacity to fertilize an oocyte, support normal embryonic development, and achieve a live birth (Wang and Swerdloff, 2014). Conventional semen parameters, such as concentration, motility, and morphology, provide descriptive information on spermatogenesis and sperm maturation but offer limited insight into sperm function. Consequently, semen analysis shows substantial overlap between fertile and infertile men and demonstrates limited diagnostic discrimination for male infertility, except at extreme values (Barratt *et al.*, 2017).

During spermiogenesis, sperm DNA undergoes extensive chromatin remodelling, with histones largely replaced by protamines, resulting in a highly condensed, stable chromatin structure that protects the paternal genome during transit and is essential for fertilization and early embryogenesis (Sakkas and Alvarez, 2010). Disruption of chromatin condensation or integrity—induced by oxidative stress, defective packaging, or abortive apoptosis—renders sperm DNA vulnerable to damage (Ribas-Maynou and Benet, 2019). Both single- and double-strand DNA breaks have been associated with impaired fertilization, abnormal embryo development, increased miscarriage risk, and adverse reproductive outcomes (Simon *et al.*, 2017). Thus, sperm DNA integrity represents a fundamental determinant of sperm functional competence that is not captured by routine semen analysis.

Sperm DNA fragmentation (SDF) testing quantifies the proportion of spermatozoa with damaged DNA and has been proposed as a functional biomarker of male fertility (Barratt *et al.*, 2017). In principle, SDF testing may detect sperm DNA damage, inform reproductive risk, and support clinical decision making. However, its clinical applicability is limited by substantial methodological and interpretative challenges. Multiple assays, including the sperm chromatin structure assay (SCSA), TUNEL, sperm chromatin dispersion (SCD), and COMET, assess different aspects of DNA damage using assay-specific methodologies and threshold cut-off values (Simon *et al.*, 2017). These thresholds vary across assays, laboratories, and populations, lack standardization, and have not been consistently clinically validated (Barratt *et al.*, 2017). Consequently, although SDF thresholds categorize levels of sperm DNA damage, they remain largely analytical rather than clinically diagnostic and do not provide a reliable basis for diagnosing male infertility or guiding personalized therapeutic interventions.

The lack of agreement on the timing of SDF testing, the appropriate assay to utilize, accepted threshold cut-off values, and the impact of the test result on management has resulted in variable clinical application and persistent ambiguity concerning its actual clinical value (Zini and Sigman, 2009; Cissen *et al.*, 2016; Esteves *et al.*, 2017; WHO, 2021).

To be considered for routine clinical practice, a test must demonstrate clinical utility, meaning it provides information that meaningfully improves patient outcomes by informing diagnosis, prognosis, or management, since biological relevance alone is insufficient (Barratt *et al.*, 2017). Clinical utility must be assessed within established test performance frameworks, namely screening, diagnostic, or prognostic testing. A screening test identifies individuals at risk within asymptomatic populations, a diagnostic test distinguishes affected from unaffected individuals, and a prognostic test predicts future outcomes to guide management (Bossuyt *et al.*, 2015). Use of a test without a clearly defined clinical objective risks misinterpretation, inappropriate escalation of treatment, and deviation from evidence-based practice. In this context, the increasing use of SDF testing in fertility clinics, despite a lack of consensus on its intended clinical role, emphasizes the necessity of a structured evaluation of its clinical utility. The objective of this opinion paper is to critically evaluate the clinical utility of SDF testing by systematically examining its performance within established screening, diagnostic, and prognostic test performance frameworks. By applying these structured criteria, we aim to clarify when, where, and how SDF testing should be used in infertility evaluation and to provide rational guidance for its appropriate use in clinical practice.

### SDF testing for male infertility as a screening test

A screening test aims to identify individuals at risk for a specific disease within an asymptomatic population, anticipating that early detection will facilitate successful intervention and quantifiable health benefits. Screening tests are not diagnostic; rather, they are intended to identify individuals who may require additional assessment. Male infertility is a clinically significant health condition. The essential characteristics of a successful screening test, comprising the significance of the condition, test efficacy, acceptability, accessibility to appropriate treatment, and tangible advantages of early detection, are delineated in the Wilson–Jungner criteria (Wilson and Jungner, 1968), and the performance of SDF as a screening test is assessed and summarized in Table 1.

**Table 1.** Criteria for a useful screening test.

Criteria	Requirement	Assessment for SDF testing	Meets criteria
Importance	Important health problem	Infertility is important; SDF is a biomarker, not a disease	◑
Natural history	Latent stage defined	Temporal behaviour and reversibility are poorly understood	✖
Biological plausibility	Disease progression known	Associations exist; causality is uncertain	◑
Test	Standardized test	Multiple assays; poor standardization	✖
Acceptability	Acceptable to the population	Minimally invasive; acceptable	✔
Accuracy	High sensitivity and specificity	Low sensitivity, modest specificity, and poor discrimination^1^	✖
Treatment	Effective treatment available	No proven outcome-modifying intervention	✖
Early benefit	Early detection improves outcomes.	No consistent evidence	✖
Policy	Clear thresholds	No agreed cut-offs or pathways	✖
Cost	Cost-effective	Not established	✖
Continuity	Suitable for repetition	High biological variability	✖

✔ Meets criteria ◑ Partially meets criteria  ✖ Does not meet key criteria.

Source: Wilson and Jungner (1968).

1 Vitthala *et al.* (2026).

SDF, sperm DNA fragmentation.

Population-based screening for male infertility is not routinely performed; instead, the examination of male reproductive function is conducted opportunistically, primarily during infertility evaluations. When assessed using the Wilson–Jungner framework, SDF testing fails to satisfy multiple essential screening criteria, notably regarding test sensitivity, standardization, accessibility of effective treatment following detection, and evidence that early identification improves reproductive outcomes (Table 1).

### SDF testing for male infertility as a diagnostic test

The primary objective of a diagnostic test is to detect the presence of disease or a clinically relevant condition with sufficient accuracy to inform diagnosis and guide management. In male infertility, the condition of interest is impaired male reproductive potential, while outcomes used to infer diagnostic performance include natural conception, miscarriage, and live birth. Diagnostic accuracy reflects the performance of the index test relative to an appropriate reference standard; at present, routine semen analysis remains the only accepted reference standard (Bossuyt *et al.*, 2003).

Several assays assess SDF, of which the SCSA, alkaline Comet, SCD, and TUNEL are the most established. These assays show population-level associations with reproductive outcomes across natural conception, IUI, IVF, and ICSI (Esteves *et al.*, 2021). Thresholds of 30% are associated with longer time to pregnancy and poorer pregnancy outcomes (Santi *et al.*, 2018; Esteves *et al.*, 2021). The diagnostic test accuracy (DTA) of SDF has been assessed using established DTA and clinical utility frameworks (Fryback and Thornbury, 1991; Bossuyt *et al.*, 2003), as summarized in Tables 2 and 3. SDF testing demonstrates modest specificity with consistently low sensitivity, resulting in weak likelihood ratios and limited diagnostic discrimination. These observations are supported by our recent systematic review and meta-analysis of 52 studies (19 930 participants), which demonstrated low sensitivity, modest specificity, weak likelihood ratios, and limited discriminatory capacity across natural conception and assisted reproductive settings (Vitthala *et al.*, 2026). Routine semen analysis is also imperfect, with reported specificity of 26–65% and sensitivity of 57–85% (Guzick *et al.*, 2001).

**Table 2.** Criteria for a useful diagnostic test framework.

Criteria	Diagnostic requirement	Assessment for SDF	Meets criteria
Clinical relevance	Clinically meaningful condition	Male infertility is relevant; SDF is a biomarker, not a disease	◑
Case definition	Clear disease definition	No distinct disease entity is defined by SDF	✖
Test purpose	Confirms/excludes disease	Cannot reliably confirm or exclude male-factor infertility	✖
Accuracy	High sensitivity and specificity	Low sensitivity, modest specificity, and weak likelihood ratios^1^	✖
Reproducibility	Consistent across settings	Inter-assay and inter-lab variability	✖
Thresholds	Stable, interpretable cut-offs	Cut-offs vary by assay and population	✖
Added value	Beyond existing tests	Limited incremental value over semen analysis	◑
Actionability	Influences diagnosis/management	Does not consistently alter diagnostic decisions	✖
Acceptability	Acceptable to patients	Minimally invasive; acceptable	✔
Feasibility	Practical for routine use	Available but uneven expertise	◑
Cost-benefit	The benefit justifies the cost	Cost-effectiveness not demonstrated	✖

✔ Meets criteria  ◑ Partially meets criteria  ✖ Does not meet key criteria.

Sources: Bossuyt *et al.* (2003); Fryback and Thornbury (1991).

1 Vitthala *et al.* (2026).

SDF, sperm DNA fragmentation.

**Table 3.** Diagnostic and prognostic performance of sperm DNA fragmentation assays for pregnancy and miscarriage outcomes.

Outcome	Assay	Sensitivity	Specificity	PPV	NPV	LR+	LR−	Remarks
**Pregnancy**								
	COMET	0.547	0.763	0.69	0.62	2.309	0.593	Natural conceptions and assisted reproductive techniques
	SCD	0.486	0.651	0.58	0.55	1.394	0.789	
	SCSA	0.36	0.764	0.6	0.54	1.525	0.838	
	TUNEL	0.369	0.745	0.59	0.54	1.445	0.847	
**Miscarriage**								
	COMET	0.542	0.778	0.7	0.62	2.44	0.589	
	SCD	0.336	0.777	0.58	0.53	1.507	0.855	
	SCSA	0.218	0.854	0.58	0.51	1.491	0.916	
	TUNEL	0.401	0.862	0.74	0.58	2.91	0.695	

PPV, positive predictive value; NPV, negative predictive value; LR+, positive likelihood ratio; LR−, negative likelihood ratio. COMET, alkaline Comet assay; SCD, sperm chromatin dispersion; SCSA, sperm chromatin structure assay; TUNEL, terminal deoxynucleotidyl transferase dUTP nick end labelling.

Data derived from Vitthala *et al.* (2026).

Despite biological plausibility, SDF testing cannot diagnose male infertility with sufficient accuracy for several reasons. First, SDF assays quantify the proportion of damaged sperm within an ejaculate rather than the functional competence or DNA integrity of individual spermatozoa, and no universally validated cut-off reliably predicts individual outcomes (Mehta, 2017). Second, SDF is a surrogate of chromatin vulnerability assessed under *in vitro* conditions and may not reflect *in vivo* function (Panner Selvam *et al.*, 2021). Third, differences in methodology, lesion detection, and assay-specific thresholds produce substantial inter- and intra-laboratory variability, undermining reproducibility and interpretability and precluding consensus on a preferred diagnostic assay (Esteves *et al.*, 2017, 2021).

Fourth, SDF testing does not specify the nature or genomic location of DNA damage, increasing the risk of false positives, and many assays do not distinguish single from double-strand breaks, which have distinct reproductive consequences, further limiting the test’s positive predictive value (Esteves *et al.*, 2021). Fifth, diagnostic performance is strongly modified by female factors, particularly the capacity of oocytes to repair DNA, resulting in higher predictive value with advanced maternal age but limited independent utility in younger women (Sakkas and Alvarez, 2010; Jin *et al.*, 2015; Liang *et al.*, 2019; Esteves *et al.*, 2020). Finally, reliance on surrogate reproductive outcomes introduces confounding by female, embryonic, and treatment-related factors.

Taken together, and consistent with Tables 2 and 3, SDF testing does not meet criteria for a standalone diagnostic test for male infertility. The low sensitivity, modest specificity, weak likelihood ratios, assay heterogeneity, and dependence on female factors and treatment modifiers limit its diagnostic reliability.

### SDF testing for male infertility as a prognostic test

The objective of a prognostic test is to estimate the likelihood of future clinical events over a defined time horizon, enabling risk stratification and selection of management strategies that may modify outcomes. In reproductive medicine, a prognostic test should reliably predict clinically meaningful outcomes, such as pregnancy, miscarriage, or live birth, and guide decisions between expectant management and assisted reproductive treatment, including IUI, IVF, and ICSI.

Prognostic performance and clinical utility of SDF testing have been evaluated using established prognostic test-performance and clinical utility frameworks, incorporating principles of discrimination, calibration, and clinical actionability (Altman and Royston, 2000; Vickers and Elkin, 2006; Steyerberg *et al.*, 2010), and are summarized in Tables 3 and 4. Men unable to achieve pregnancy for more than 1 year have ∼2-fold higher SDF levels than fertile men (Zini *et al.*, 2008), and elevated SDF has been associated with reduced pregnancy rates following IUI and assisted reproductive technologies, including ICSI (Boe-Hansen *et al.*, 2006). Although elevated SDF is associated with adverse reproductive outcomes at the population level, hierarchical modelling reveals substantial heterogeneity across assays and thresholds, with limited individual-level predictive performance, suggesting caution in routine practice (Vitthala *et al.*, 2026).

**Table 4.** Criteria for a useful prognostic test framework.

Criteria	Prognostic criteria	Assessment for SDF testing	Meets criteria
Clinical relevance	Predicts meaningful future outcomes	Outcomes include pregnancy, miscarriage, and live birth.	✔
Outcome definition	Clearly defined and measurable outcomes	Outcomes are defined as couple-dependent and multifactorial.	◑
Discrimination	Separates high- vs low-risk individuals.	Poor-to-moderate discrimination with substantial overlap	✖
Sensitivity	Identifies those at risk	Consistently low sensitivity across natural conception and ART	✖
Specificity	Identifies those without risk	Modest specificity; insufficient for individual-level prediction	◑
Likelihood ratios	Clinically useful post-test probability change	Weak likelihood ratios with limited clinical impact	✖
Threshold stability	Stable, outcome-relevant cut-offs	Thresholds vary by assay, population, and outcome.	✖
Reproducibility	Consistent across settings	Inter-assay and inter-laboratory variability	✖
Independence	Independence of major confounders	Strongly modified by female age, oocyte quality, and ART technique	✖
Actionability	Alter treatment or counselling	It does not reliably change management decisions but may be useful in unexplained infertility, RIF, and RPL.	◑
Added value	Improves prediction beyond standard factors	Limited incremental prognostic value. May be useful in unexplained infertility/RIF and RPL.	◑
Clinical utility	Demonstrates net benefit in practice	No consistent evidence of outcome improvement. It may be useful in unexplained infertility/RIF and RPL.	✖

✔ Meets criteria  ◑ Partially meets criteria  ✖ Does not meet key criteria.

Prognostic test-performance criteria are derived from established principles of prognostic model evaluation and clinical utility assessment (Altman and Royston, 2000; Vickers and Elkin, 2006; Steyerberg *et al.*, 2010). RIF, recurrent implantation failure; RPL, recurrent pregnancy loss; SDF, sperm DNA fragmentation.

Despite this association, SDF does not meet the criteria for a useful prognostic test (Table 4). First, it lacks forward-looking capacity: most men presenting to fertility clinics have already elevated SDF, limiting the prediction of future risk. Second, the underlying aetiopathogenesis is multifactorial, involving defective chromatin condensation, direct oxidative DNA damage, and downstream oxidative stress; oxidative stress acts as both cause and consequence, while abnormal packaging increases susceptibility to damage (Gosálvez *et al.*, 2015; Panner Selvam *et al.*, 2021). Routinely used assays (SCSA, Comet, SCD, TUNEL) primarily capture direct DNA damage but incompletely assess chromatin defects, limiting biological completeness.

Third, actionability is restricted. Interventions that reduce SDF apply mainly to selected aetiologies (e.g. varicocele, lifestyle factors, infections, and certain exposures), whereas options are limited in unexplained infertility, recurrent pregnancy loss, and recurrent ART failure (Esteves *et al.*, 2021). Current management relies largely on non-specific antioxidants without routine oxidative assessment, yielding inconsistent benefit, particularly in cases of unexplained infertility and recurrent ART failure where targeted treatments are lacking (Shamsi *et al.*, 2011; Esteves *et al.*, 2020).

Consistent with Tables 3 and 4, SDF testing shows poor discrimination, low sensitivity at clinically relevant thresholds, weak likelihood ratios, and substantial overlap in outcomes across natural conception, IUI, IVF, and ICSI. Negative results (low SDF levels) provide limited reassurance, and positive results (elevated SDF levels) do not consistently change management. Prognostic performance is further modified by female factors, particularly age and ovarian reserve, both of which determine oocyte DNA repair capacity.

Taken together, although elevated SDF correlates with adverse outcomes at a population level, limited discrimination, unstable thresholds, restricted actionability, and dependence on female factors and treatment modifiers preclude its use as a reliable prognostic test for male infertility.

## Discussion

The clinical value of any test depends on a clear understanding of its intended purpose. Screening tests aim to identify disease risk in asymptomatic populations, diagnostic tests distinguish affected from unaffected individuals, and prognostic tests predict future outcomes to guide management. Applying these principles to SDF testing highlights important limitations that constrain its current role in male infertility, such as its inability to provide a definitive diagnosis of infertility or predict treatment success based solely on SDF.

From a screening perspective, SDF testing does not meet fundamental requirements. Male infertility is not subject to population-based screening, and SDF lacks the sensitivity, standardization, defined target population, and demonstrable outcome benefit required for use in asymptomatic individuals. In addition, the predictive value of any test depends on disease prevalence. The prevalence of male infertility in the general population is unknown and strongly influenced by female reproductive factors, so the predictive performance of SDF outside infertile cohorts cannot be meaningfully defined. Consequently, SDF cannot be justified as a screening investigation.

SDF is not a reliable diagnostic test for distinguishing infertile from fertile males. Diagnostic accuracy studies and meta-analyses generally demonstrate low sensitivity, moderate specificity, weak likelihood ratios, and poor-to-moderate discrimination in both natural conception and assisted reproductive contexts. Despite conventional semen analysis being an imperfect reference standard, SDF offers minimal additional diagnostic value, while assay heterogeneity, unstable cut-off levels, and inter-laboratory variability further compromise dependability.

The prognostic performance of SDF testing is limited and clinical situation dependent. Although elevated SDF is associated with adverse reproductive outcomes at a population level, its ability to predict individual outcomes such as pregnancy, miscarriage, or live birth remains poor, with low sensitivity at commonly used thresholds, weak likelihood ratios, and substantial overlap between outcome groups across natural conception, IUI, IVF, and ICSI. Female factors, particularly the ability of oocytes to repair DNA and maternal age, further confound prognostic interpretation, making it difficult for clinicians to accurately assess the impact of SDF on individual reproductive outcomes. Clinically, SDF is most informative when it prompts targeted, aetiology-specific interventions. Evidence related to the selective use of testicular sperm for ICSI in men with persistently elevated SDF and unexplained ART failure or recurrent pregnancy loss remains inconsistent and methodologically limited. Some research suggests that reproductive outcomes may be better, but most of the data are observational, varied, and potentially biased because they fail to adjust adequately for confounders, particularly female factors. Sample sizes are often small, and there is a lack of sufficiently powered randomized trials. As a result, any stated advantages should be approached with caution (Moskovtsev *et al.*, 2012; Simon *et al.*, 2017; Ribas-Maynou and Benet, 2019; Alharbi *et al.*, 2020; Romano *et al.*, 2023; Humaidan *et al.*, 2026).

SDF reflects biologically relevant sperm genomic integrity but does not support routine use as a screening, diagnostic, or prognostic test in the general infertile population. Across natural conception and assisted reproductive settings, SDF testing demonstrates limited sensitivity, modest specificity, low likelihood ratios, substantial assay heterogeneity, and variable thresholds, resulting in poor-to-moderate discriminatory performance at the individual level. While increased SDF may be associated with negative reproductive outcomes in certain clinical scenarios, such as unexplained infertility, recurrent implantation failure, and recurrent pregnancy loss, these correlations primarily derive from observational studies with methodological limitations and confounding by female and treatment-related factors. Associations are not causes, and this distinction must be emphasized. There is still insufficient evidence to justify modifying treatment techniques, such as using testicular sperm for ICSI, in the absence of well-designed, adequately powered randomized trials. Selective testing in clearly defined subfertility populations may provide additional information; however, its use should be limited to indication-based or prospective research settings until validated through standardized methodologies and supported by robust therapeutic evidence. The routine incorporation of SDF testing into fertility evaluation should therefore be carefully considered. A selective, indication-based approach, with transparent counselling on limitations and uncertain incremental benefit, may represent a balanced strategy.

According to ESHRE and ASRM/AUA guidelines (ASRM, 2020; AUA/ASRM, 2020; ESHRE, 2023), SDF testing should not be used regularly or as a stand-alone diagnostic tool. Instead, it should only be used in certain infertile subpopulations, such as those with unexplained infertility, recurrent implantation failure, and recurrent pregnancy loss. Even in these cases, the limitations of SDF as a test must be acknowledged until better-quality data from appropriate research studies are available.

## Conclusion

When evaluated systematically as a screening, diagnostic, and prognostic test, SDF testing fails to meet the core performance requirements expected of any of these categories. It does not meet population-level screening criteria, lacks sufficient accuracy and standardization to serve as a standalone diagnostic test, and demonstrates limited discriminative and actionable performance as a prognostic tool. While SDF captures a biologically relevant aspect of sperm genomic vulnerability, its clinical utility is constrained by assay heterogeneity, unstable thresholds, modest test-performance metrics, and strong dependence on female and treatment-related modifiers. Consequently, SDF testing should not be incorporated into routine fertility evaluation as a universal screening, diagnostic, or prognostic investigation. If used in any clinical scenario, its limitations should be highlighted to stakeholders, and test results should be interpreted with caution, particularly because SDF testing may not accurately reflect overall fertility potential and can lead to misinformed treatment decisions.

Where SDF testing is considered, it should be reserved for clearly defined clinical scenarios, such as unexplained infertility, recurrent implantation failure, or recurrent pregnancy loss, and used as one component of a comprehensive male fertility assessment rather than as a standalone decision-making tool. Advancing the clinical utility of SDF testing will require future research focussing on assay standardization, clinically validated thresholds, prespecified patient subgroups, and adequately powered prospective studies to assess whether SDF-guided interventions improve live birth rates or reduce miscarriage rates.

## Data Availability

No new data was generated or analysed in support of this Opinion article. All data referenced are derived from previously published studies cited in the reference list.
